# Risk Factors for Obstructive Sleep Apnea Syndrome in Children: State of the Art

**DOI:** 10.3390/ijerph16183235

**Published:** 2019-09-04

**Authors:** Giampiero Gulotta, Giannicola Iannella, Claudio Vicini, Antonella Polimeni, Antonio Greco, Marco de Vincentiis, Irene Claudia Visconti, Giuseppe Meccariello, Giovanni Cammaroto, Andrea De Vito, Riccardo Gobbi, Chiara Bellini, Elisabetta Firinu, Annalisa Pace, Andrea Colizza, Stefano Pelucchi, Giuseppe Magliulo

**Affiliations:** 1 Department of “Organi di Senso”, University “Sapienza”, 00185 Rome, Italy; 2 Department of Head-Neck Surgery, Otolaryngology, Head-Neck and Oral Surgery Unit, Morgagni Pierantoni Hospital, 47121 Forlì, Italy; 3 Ear-Nose-Throat & Audiology Unit, University of Ferrara, 44121 Ferrara, Italy; 4 Department of Oral and Maxillo Facial Sciences, University “Sapienza”, 00185 Rome, Italy

**Keywords:** pediatric OSAS, obesity, adenotonsillar hypertrophy, craniofacial abnormalities, allergic rhinitis, inflammation

## Abstract

The obstructive sleep apnea syndrome (OSAS) represents only part of a large group of pathologies of variable entity called respiratory sleep disorders (RSD) which include simple snoring and increased upper airway resistance syndrome (UARS). Although the etiopathogenesis of adult OSAS is well known, many aspects of this syndrome in children are still debated. Its prevalence is about 2% in children from 2 to 8 years of age, mostly related to the size of the upper airways adenoid tissue. Several risk factors linked to the development of OSAS are typical of the pediatric age. The object of this paper is to analyze the state of the art on this specific topic, discussing its implications in terms of diagnosis and management.

## 1. Introduction

Respiratory sleep disorders (RSD) in children are characterized by a variable obstruction of the upper airway and different degrees of alteration in gas exchange during the night [1,2]. 

The third edition of the International Classification of Sleep Disorders (ICSD-3) defines OSAS as a polysomnography (PSG)-determined obstructive respiratory disturbance index (RDI) ≥ 5 events/h associated with the typical symptoms of OSAS (e.g., unrefreshing sleep, daytime sleepiness, fatigue or insomnia, awakening with a gasping or choking sensation, loud snoring, or witnessed apneas), or an obstructive RDI ≥ 15 events/h (even in the absence of symptoms) [3]. In addition to the apneas and hypopneas that are included in the Apnea-Hypopnea Index (AHI), the RDI includes respiratory effort-related arousals (RERAs). The scoring of respiratory events is defined in the AASM Manual for the Scoring of Sleep and Associated Events: Rules, Terminology and Technical Specifications, Version 2.3 (AASM Scoring Manual) [4]. However, some variability in the definition of a hypopnea event should be noted. 

According to the AASM Scoring Manual recommended definition, changes in flow should be associated with a 3% oxygen desaturation or a cortical arousal, although an alternative definition that requires association with a 4% oxygen desaturation without consideration of cortical arousals is accepted. Depending on which definition is used, the AHI may be considerably different in any given individual [5,6,7]. The discrepancy between these and other hypopnea definitions used in research studies makes the evaluation of evidence regarding the diagnosis of OSAS a complex matter.

The apnea-hypopnea index (AHI) is the average number of disordered breathing events per hour. Typically, the OSA syndrome is defined as an AHI of 5 or greater. An AHI of 5–15 is considered as mild, 15–30 is moderate and more than 30 events per hour characterizes severe sleep apnea.

The precise incidence of OSAS in adults is still unknown. It is estimated that 24 percent of men and 9 percent of women have the breathing symptoms of OSAS with or without daytime sleepiness, but about 80 percent of adults with OSAS remain undiagnosed [8].

On the other hand, respiratory sleep disorders (RSD) in children are characterized by a variable obstruction of the upper airway and different degrees of alteration in gas exchange during the night. 

The clinical presentation goes from habitual snoring to complete obstruction of the airway [9,10]. The European Respiratory Society (ERS) taskforce for the diagnosis and management of obstructive RSD in childhood defines OSAS as “a syndrome of upper airway dysfunction during sleep, characterized by snoring and/or increased respiratory effort secondary to increased upper airway resistance and pharyngeal collapsibility” [11]. In childhood OSAS there is not a clear correlation between the severity of the clinical presentation and daytime symptoms, with a more nuanced range of symptoms [12].

Gozal et al. [13] proposed several criteria, classified into major and minor, for the diagnosis of OSAS in children and, above all, to assess the need for treatment. The major ones include an AHI > 2, RDI > 2, Nadir SpO2 < 90%, excessive daytime sleepiness, academic difficulties, hyperactive behavior, hypertension, enuresis, and obesity. Among the minor ones, there are high levels of CRP, LDL, fasting insulin and low levels of HDL, recurrent middle ear otitis and adenotonsillar grade >1. The positivity of five major criteria, or three major criteria plus 3 minor criteria, indicates the need for therapeutic procedures.

According to latest guidelines, the general incidence of OSAS in the pediatric population is about 2% [9]. Most children are around 2–8 years of age, due to the relative size of the lymphatic tissue of the upper airways [14]. OSAS is more common in males than females. African Americans and obese children are both at increased risk for OSAS [15]. While many studies have addressed the etiopathogenesis of adult OSAS, many aspects of this syndrome in children remain unclear. However, there are many risk factors which can lead to a reduction or collapse of the upper airways and which may contribute to the pathogenesis of OSAS.

The diagnosis of pediatric OSAS include a detailed clinical history, focusing on physical findings, especially in the ENT district, nocturnal and diurnal symptoms and comorbidities followed by specific questionnaires administered to parents. Nocturnal oximetry and ambulatory polysomnography are mandatory. In patients with residual OSAS after surgery, DISE should be considered to detect the anatomical site of potential collapse.

To our knowledge, few papers have been published on the risk factors linked to OSAS, especially in the pediatric age [9,16]. We were able to find out 182 articles from 2012 to 2019 on Pubmed and MEDLINE using the keywords “Risk factors; Pediatric OSAS; Obesity; Adenotonsillar hypertrophy; Craniofacial abnormalities; Allergic rhinitis; Inflammation”. Only 123 articles discussed this specific topic in children. Furthermore, it should be noted that the majority of the papers analyzed only one single risk factor and rarely multiple factors, focusing essentially on the overview of the OSAS. The very exhaustive papers published by Marcus C.L. et al. [9] and Li Z. et al. [16] are typical examples of this observation. They made a general, wonderful presentation of the pathology, but dedicated just a few paragraphs in evaluating the various risk factors. 

Thus, the aim of this paper is devoted to analyzing the state of the art on this specific and its impact on diagnosis and treatment.

## 2. Risk Factors

### 2.1. Obesity

Obesity is one of the most important risk factors for OSAS in both the adult and pediatric populations.

Obesity affects OSAS mainly through two mechanisms: The presence of fat at the level of the pharyngeal soft tissue reduces the caliber of the lumen and increases the collapse of the structures themselves. Secondly, the increased presence of fat in the thoracic and abdominal walls significantly reduces respiratory function in these patients [17,18].

Each increment in body mass index (BMI) above the 50th percentile is associated with around a 10% increased risk for OSAS [19].

From 1975 to 2016, the mean BMI in children and adolescents increased globally, up to 18.6 kg/m^2^ (18.4–18.7) for girls and 18.5 kg/m^2^ (18.3–18.7) for boys in 2016. Moreover, childhood obesity is not only a widespread phenomenon, but also a persistent one: About 50% of obese children are also likely to be obese adults [20].

Overweight or obese children have a higher risk of developing OSAS compared with normal-weighted children [21]. 

Even though adenotonsillectomy (AT) represents the first line therapy for these children, several studies have reported that obesity increases the risk of persistent OSAS after surgery [22,23,24,25]. To this regard Xu Z. et al. reported a direct correlation between the body mass index standard deviation score (BMI SDS) and the apnea-hypopnea index (AHI) [26]. 

Weight-loss management could be the key in the treatment of obesity-related OSAS in children and adolescents; specially in those that have previously performed adeno-tonsillectomy surgery without clinical results. Despite many studies supported weight-loss management as a key point therapy for OSAS in obese adults, only a few have studied the effect of obesity treatment on childhood OSA [27,28,29]. Andersen I.G. et al. [30] conducted a prospective longitudinal study on a population of 62 children and adolescents treated in a chronic care multidisciplinary overweight- and obesity treatment clinic. They observed a normalization of AHI in 38% of them after 6 months of obesity treatment and in 44% after one year of weight-loss.

These evidence lead us to consider weight-loss as first line therapy for these children with elevated BMI.

Studies that have analyzed obesity as pediatric OSAS risk factor are reported in Table 1.

### 2.2. Adenoid and/or Tonsil Hypertrophy

The lymphoid tissue of the Waldeyer ring is more developed at an age between 3 and 6 years, according to the peak incidence of OSAS [31,32]. Adenoid and/or tonsil hypertrophy are the most common causes of upper airway lumen reduction in children, evaluated by the Friedman Grading Scale (Table 2). 

**Figure 1:**
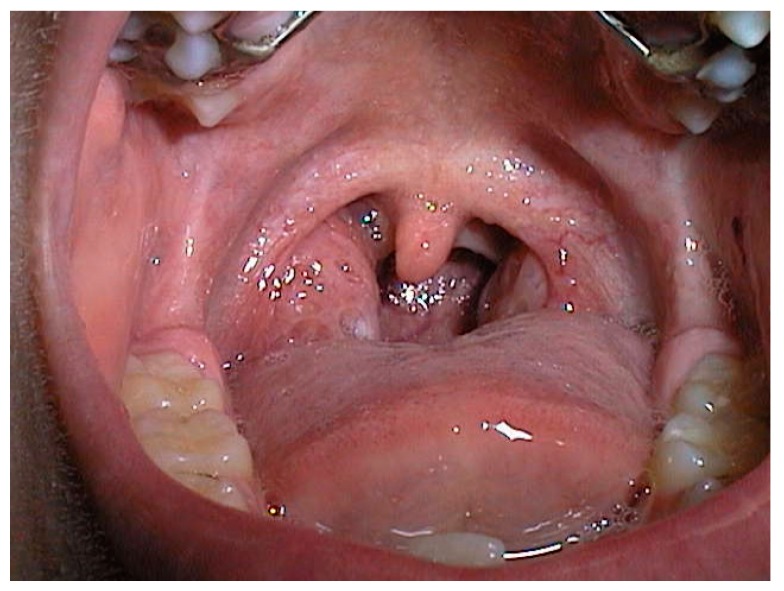
Grade III tonsil hypertrophy in a 6 year-old child. Courtesy of Professor C. Vicini—Department of Head-Neck Surgery, Otolaryngology, Head-Neck and Oral Surgery Unit, Morgagni Pierantoni Hospital, Forlì, Italy.

Adenotonsillar hypetrophy contributes to the narrowing of the retro-palatal area, which has the smallest cross-sectional area and is therefore the most frequent site of obstruction, as shown in a 6 years old patient in Figure 1. In particular, children with Grade IV tonsils (kissing tonsils) are very susceptible to developing sleep disorders, due to oropharyngeal narrowing and lateral collapse. 

Adenotonsillar hypertrophy leads to mouth breathing, nasal congestion, hyponasal speech, snoring, chronic sinusitis, and recurrent otitis media, as well as poor brain development and emotional disturbances. Kurnatowski P. et al. [33] in their study of six- to nine-year-old children reported high scores of emotional instability in the adenotonsillar hypertrophy group, compared to healthy controls, with good results on the behavior of most of the children 3–10 months after surgery. Some authors highlighted the role of inflammation in the development of adenotonsillar hypertrophy and OSAS, given the increased expression of various mediators of inflammatory responses in the tonsils and improvement following treatment with anti-inflammatory agents such as corticosteroids, suggesting a multi-disciplinary approach for treatment.

The gold standard of surgical treatment is represented by adenotonsillectomy [9,34]. Brietzke et al. [34] demonstrated the resolution of the polysomnographic findings in 83% of children with OSAS without other co-morbidities. Several recent studies have confirmed the efficacy but have suggested long-term follow up, especially in children with concomitant comorbidities, in order to avoid residual OSAS [35,36]. On the other hand, in the adult OSAS population, tonsillectomy is more often associated with palatal surgery. However, Senchak AJ et al. [37] proposed tonsillectomy as isolated surgery. They enrolled 202 subjects. The AHI before surgery ranged from 5.4 to 56.4 events per hour. The mean AHI decreased from 18 to 3.2 events per hour, a reduction of 82%. Following tonsillectomy alone, there were statistically significant reductions in median RDI and in the Epworth Sleepiness Scale (ESS) and Berlin scores. They drew the conclusion that adult tonsillectomy alone could be effective, especially in young overweight men with large tonsils, moderate OSAS and low Friedman stage.

**Figure 2:**
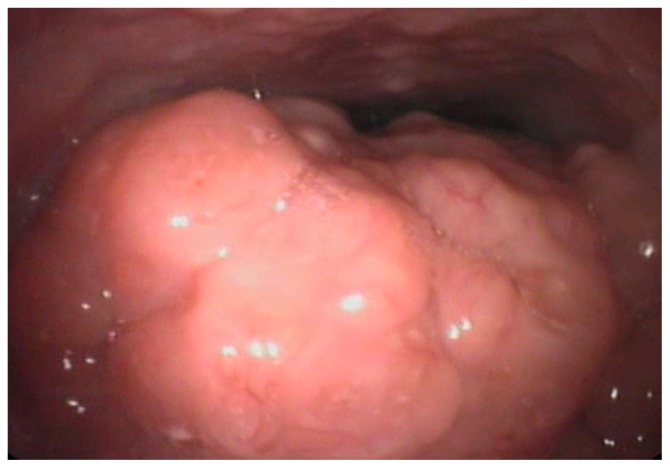
Lingual tonsil hypertrophy in a 11 year-old child. Courtesy of Professor C. Vicini—Department of Head-Neck Surgery, Otolaryngology, Head-Neck and Oral Surgery Unit, Morgagni Pierantoni Hospital, Forlì, Italy.

**Figure 3:**
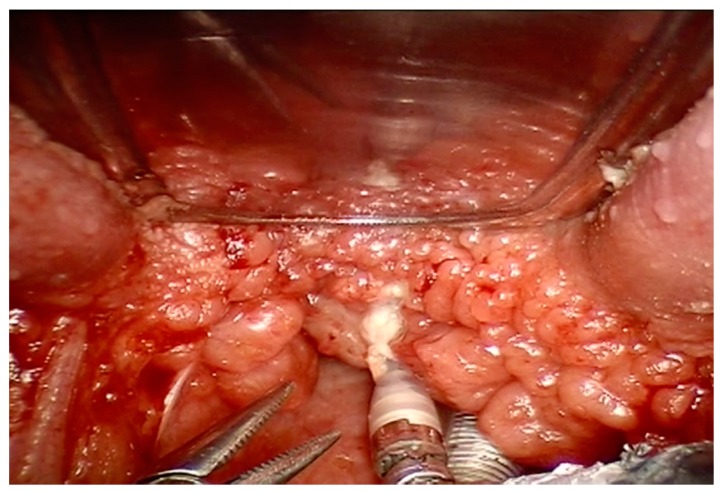
Transoral robotic surgery (TORS) to remove an exuberant lingual tonsil in a 12-year-old child. Courtesy of Professor C. Vicini—Department of Head-Neck Surgery, Otolaryngology, Head-Neck and Oral Surgery Unit, Morgagni Pierantoni Hospital, Forlì, Italy.

Recent studies have also suggested the role of lingual tonsil hypertrophy in the pathogenesis of pediatric OSAS, as frequent evidenced in the adult population [8,9,10,11,12]. Figure 2 shows lingual hypertrophy in a 11-year-old child. In adults, the procedure of reducing lingual tonsil through Trans Oral Robotic Surgery (TORS) is widely used. In children, it is not a common procedure [38,39]. In particular, the young patient of Figure 3 underwent robotic surgery due to severe OSAS with disabling symptoms without adenotonsillar hypertrophy or other risk factors.

The possibility of intracapsular tonsillotomy using Debrider or Coblation should be considered in this type of surgery in order to remove only the exuberant lymphatic tissue of tonsils. Reported similar intracapsular tonsillotomy seems to show similar results in terms of AHI reduction with respect classical extracapsular tonsillectomy with a lower risk of postoperative bleeding [40].

Supraglottoplasty in cases of laryngomalacia associated with exuberant arytenoid tissue or epiglottoplasty in case of epiglottis hypertrophy/instability, are possible treatment options and should always be considered [37,38].

Midline posterior glossectomy is less frequently performed in pediatric OSAS patients [41].

Studies that have analyzed adenoid and/or tonsil hypertrophy as a pediatric OSAS risk factor have been reported in Table 3. 

### 2.3. Allergic Rhinitis

It is believed that allergic rhinitis (AR) can affect sleep through different mechanisms. Nasal congestion secondary to the nasal mucosa inflammatory process induces increased airway resistance and may result in oral breathing, sleep disruption, and fatigue [42]. 

Nasal patency is mainly regulated by the capacitance vessels of the middle and inferior turbinates [43]. What happens in an allergic patient is an increased nasal resistance with dilatation of these capacitance vessels, with mucosal edema and mucous secretions [44]. The most common symptoms of allergic rhinitis patients are rhinorrhea, nasal blockage or congestion, and stuffy nose. Clearly, nasal obstruction is also associated with olfactory disorders because of the reduction of odourants that enter the nose during breathing [45,46].

Further exacerbating airflow limitation, an increase in airflow velocity may cause paradoxical airway narrowing in the nasal valve and, as the obstruction progresses, in the oropharynx too.

Lofaso et al. [47] used posterior rhinomanometry to highlight the correlation between nasal obstruction and OSAS severity, independently of other risk factors. They evaluated 541 unselected consecutive snorers referred for suspected breathing disorders during sleep over a 2 year period. In addition, cephalometric landmarks and body mass index (BMI) were obtained. Polysomnography was used to determine the number of abnormal respiratory events that occurred during sleep. They concluded that permanent physical nasal obstruction unresponsive to nasal decongestants, and functional nasal obstruction responsive to nasal decongestants, such as those that occur during vasomotor rhinitis, may contribute to sleep-disordered breathing. and is an independent risk factor for OSAS.

McNicholas WT [48] et al. demonstrated longer and more frequent obstructive apneas in patients with allergic rhinitis to ragweed, especially during the ragweed season rather than after the pollen season [47]. Furthermore, an elevated Mallampati score seems to have a synergistic effect in these patients.

Liistro et al. [49] proposed a two-hit phenomenon, with a higher relative risk for OSA in those with a reduced posterior pharynx space due to tongue base hypertrophy and nasal obstruction, compared to those with only one of these risk factors.

Additionally, inflammatory mediators of the allergic process, such as histamine and certain cytokines, may act directly on the central nervous system by altering sleep rhythm. It has been recently observed in children with sleep disorders, that the presence of allergic rhinitis (without obstructive sleep apnea) decreases REM (Rapid Eye Movement) sleep time [50]. A recent meta-analysis from Cao Y. et al. [51] evaluated the association between allergic rhinitis and obstructive sleep apnea in both adult and pediatric population. They included 44 studies, comprising 6086 participants. They reported an incidence of children diagnosed with AR 2.12 times higher in OSA/RSD patients. However, the same trend was not found in the adult population. The possible cause is related to the immaturity of the immune system in children. Medical treatment, such as nasal steroids and oral montelukast has been studied as a treatment option for OSAS children with concomitant allergic rhinitis and asthma. Kheirandish-Gozal et al. [52] described an overall success rate of 80% in children aged 2–14 years using intranasal steroid therapy and oral montelukast. The same group recently compared the effect of 16 weeks of oral montelukast therapy with placebo for treatment of childhood OSAS with good results [53]. 

More recently, the American Academy of Pediatrics stated that nasal steroid therapy is indicated for children with mild OSAS who cannot undergo surgery or for those with persisting residual OSAS after surgery [54].

Studies that have analyzed allergic rhinitis as a pediatric OSA risk factor have been reported in Table 4. 

### 2.4. Craniofacial Abnormalities and Genetics

Craniofacial abnormalities can also be a cause of upper airway obstruction syndrome. Alterations of the size, position, and geometry of the mandible and of the tongue can lead to the thickening of the retro-palatal region which, as already mentioned above, is the most frequent site of obstruction in pediatric patients. [55,56]. 

The exact role of genetics in the pathogenesis of pediatric OSAS is still a matter of debate. What is known is that some clinical syndromes such as Down, Prader–Willi and Beckwith–Wiedemann are strongly associated with OSAS. Studies focus mostly on gene polymorphisms, for example, the ApoE4 allele, TNFa 308G gene polymorphism, NADPH polymorphism [57,58,59,60].

Zaffanello M. et al. [61] reported other genetic syndromes that could be cause of OSAS: Achondroplasia [62,63,64,65], Ehlers–Danlos Syndrome [66,67], Pierre Robin sequence/complex [68], Ellis–van Creveld Syndrome [69], sickle cell disease [70] and Noonan Syndrome [71]. Any congenital or acquired conditions that involve the respiratory control center may potentially lead to the development of OSAS. Myelomeningocele, Arnold Chiari malformation, and brain injuries from trauma, tumors, surgery can be other causes of pediatric OSAS [72,73]. In such patients, surgical treatment often represents the only option possible. Maxillo-mandibular advancement (MMA) is one of the most common type of surgical procedures performed in these cases. It provides an enlargement the nasopharynx, oropharynx, and hypopharynx by advancing the soft palate and tongue and shrinking the lateral pharyngeal walls.

Saxby et al. [74] evaluated maxillomandibular advancement surgery as the gold standard for children affected by syndromic craniofacial abnormalities. The procedure showed an improvement of the Apnea-Hypopnea Index score and OSA grading in most of their 65 patients, although measures of oxygenation revealed no differences.

On the other hand, in the adult population, the indication for mid-face advancement should take into account the complication rate and cosmetic sequelae. For this reason, it is not considered as a first line option in children without facial abnormalities [75].

Orthodontic treatment represents a valid alternative in patients only presenting high-arched or narrow palates, susceptible to OSAS. The use of rapid maxillary expansion, an appliance to widen the palate and flatten the palatal arch, has shown very good polysomnographic outcomes in a recent meta-analysis [76]. Villa et al. [77] proposed oral appliance therapy for children presenting moderate to severe OSAS with malocclusion. 

Studies that have analyzed craniofacial abnormalities and genetics as pediatric OSA risk factor have been reported in Table 5.

### 2.5. Inflammatory Factors and Biomarkers

It is believed that inflammation and OSAS are strongly related. In fact, systemic inflammatory markers level such as NF-κB, C-reactive protein (CRP), TNF-a, IL-6, IL-8 IL-1a, IL-1b, and IFN-g are upregulated in subjects with OSAS. On the other hand, the concomitant reduction of anti-inflammatory factor IL-10 suggests a tilted proinflammatory state in OSAS [78,79,80].

Several studies have highlighted the role of oxidative stress in the genesis of endothelial damage in both adult and pediatric OSAS patients [81,82]. Intermittent hypoxemia leads to anoxia and re-oxygenation, which triggers the production of oxygen radicals and consequently local and systemic inflammation.

Many studies have suggested the involvement of the NF-κB-related inflammatory pathway in the pathogenesis of OSAS. The upregulation of NF-κB increases the expression of pro-inflammatory mediators and cytokines (TNF- α, IL-6, and CRP), resulting in blood vessel endothelial damage and systemic inflammation. It is widely accepted that a correlation exists between systemic inflammation and the progression of atherosclerosis and coronary artery disease in adults.

Although in adults the association between inflammation and OSA is well known, in children it is still under debate. In children, inflammatory markers (local and systemic) and proinflammatory cytokines are upregulated, which further advances lymphoid tissue proliferation. Many studies are being conducted on the use of biomarkers for the diagnosis of OSAS, based on the high levels of pro-inflammatory factors.

Biomarkers are non-invasive and simple tests to integrate diagnosis. Kallikrein-1, orosomucoid-1, uromodulin and urocotin-3 are those most frequently used. In fact, their relevance lies on the diagnostic precision in finding children affected by OSAS [83,84]. Other biomarkers less used are: Serum alpha amylase level, lipocalin-type prostaglandin D synthase, cysteinyl leukotrienes, and urinary 8-isoprostane [85,86,87,88,89]. Although the results seem promising, unfortunately these tests have shown an unreliable specificity and sensitivity. In fact, chronic inflammation can be typical of patients affected by OSAS (both adults and children) or may develop later on. Therefore, biomarkers cannot be used as predictors. For this reason, they should be considered by physicians only as an integration of the data coming from clinical evaluation and PSG.

Studies that have analyzed inflammatory factors and biomarkers as pediatric OSAS risk factor have been reported in Table 6. 

## 3. Clinical Presentation

The clinical presentation of a child with OSAS is quite suggestive and can be divided into physical findings and symptoms. At general examination, these patients present obesity, long face syndrome, craniofacial alterations, and elevated systemic blood pressure. Moreover, the ENT physical examination may frequently show adeno-tonsillar hypertrophy, inflammation of the nasal mucosa, deviation of the nasal septum, hypertrophy of the inferior turbinates, ogival palate, or macroglossia. [90,91].

The symptoms are classically divided into nocturnal and diurnal. Among the latter, the most frequent are daytime sleepiness, recurrent headache, nasal speech, hyperactivity, inattention, depression, mood instability, irritability, and aggressiveness [92,93].

Nocturnal symptoms include snoring, witnessed apnea, oral breathing, paradoxical thoracic movements, nightmares, sleepwalking, and nocturnal enuresis [94]. The well-known long-term complications in the adult population should also be mentioned, because they may also occur in the OSA pediatric population. In fact, several studies have shown an increased risk of cardiovascular and pulmonary complications, such as pulmonary hypertension, or pulmonale and right heart failure. Indeed, high levels of CRP, brain natriuretic peptide, adhesion molecules, myeloid-related protein 8/14, fatty acid-binding protein, and other factors resulting from vascular injury and endothelial activation have all been demonstrated in children with OSAS [95,96].

Moreover, children affected by severe OSAS may develop an early metabolic syndrome. This critical condition is characterized by a cluster of pathologies: Obesity, insulin resistance, systemic hypertension, and dyslipidemia. Although the association is still not clear, several studies have highlighted the link between sleep fragmentation and intermittent hypoxia in obese children and down-regulated insulin sensitivity [97,98,99,100,101].

## 4. Diagnosis

The diagnosis of pediatric OSAS is a process composed of several steps. In children the clinical presentation is more nuanced and therefore simple clinical evaluation is often misleading. The clinical history of patients with suspected OSAS is reconstructed by administering specific questionnaires to their parents. The role of the questionnaires and their effectiveness in the diagnostic process are currently under study. In particular, of all those developed, six are the most interesting and most frequently used in clinical practice.

The Pediatric Sleep Questionnaire (PSQ) is the most widely used survey consisting of 22 close-ended questions. 

The current guidelines of the European Respiratory Society Task Force define PSQ as a good test for identifying children affected by OSAS with an apnea-hypopnea index (AHI) > 5 [102].

Other less frequently used questionnaires are: Sleep clinical record (SCR) [103], OSA-18 [104], the Broullette score (BS), and I’M SLEEPY [105].

The latest and newest one is the Sleeping Sleepless Sleepy Disturbed Rest questionnaire (SSSDR). Presented in Rome in 2018, it is not included in the latest guidelines but seems to be very promising [106].

The work of Burghard et al. [107] has summed up these questionnaires. Although very useful, they still need more studies in order to be improved or to further diagnostic steps. Following the assessment of the medical history and questionnaires, the general and ENT examination is of primary importance. The general physical examination evaluates the following parameters: Height and weight growth, neuro-behavioral factors, cardiological and pneumological conditions, and eventual co-morbidities. Therefore, the collaboration between several specialists appears to be fundamental. The ENT physical examination includes an accurate assessment of the upper airway. Adenoid hypertrophy is assessed by nasal endoscopy. There are conflicting opinions on what the role of isolated adenoid hypertrophy in the genesis of OSAS may be. In fact, many studies show that adenoid hypertrophy is not sufficient to determine OSAS, but it can worsen its severity in children with other risk factors [108]. As already mentioned, tonsillar hypertrophy is one of the most frequent causes of OSAS in otherwise healthy children and there is also a correlation between tonsillar grade and OSAS severity [109].

However, not all children with marked tonsillar hypertrophy are affected by OSAS. Thus, there is an individual variability regarding the upper airway muscle tone. Particularly in obese children and in those suffering from neuromuscular diseases, tonsillar hypertrophy does not play a significant role [110].

The presence of dento-facial anomalies also belongs to the typical presentation of an OSA child. For this reason, the ENT specialist should also involve the orthodontist who, by assessing the extra and intra-oral morphological characteristics, can select patients suitable for conservative treatment. However, recent studies have shown that the adenotonsillectomy is capable of restoring correct maxillo-mandibular morphology, thus supporting the thesis that dento-facial skeletal anomalies are more a consequence of OSA than the cause [111].

Nocturnal oximetry is the most widely used screening technique for children with a strong clinical suspicion of pediatric OSAS. In fact, it has a low cost and is easy to carry out and interpret. Although its positivity permits the diagnosis of OSAS, its negativity does not exclude the pathology. According to Kirk et al. [112], the sensitivity and specificity of oximetry for the identification of moderate OSAS (AHI > 5/h), compared to the laboratory PSG test, were 67% and 60%, respectively. These authors also noticed that the oximetry algorhythm tended to overestimate the ODI at low levels and underestimate at high levels. For that reason, oximetry alone was not adequate for the diagnosis of OSAS.

Ambulatory polysomnography is the monitoring of cardiorespiratory features during sleep performed at the patient’s home. Despite the high sensitivity and specificity of the examination carried out on adult patients, its effectiveness in children is strongly related to their age. In fact, although there are several studies which validate its efficacy in school-aged children, there are a few discrepancies in the data regarding younger children [113]. Zucconi et al. [114] proposed a home portable system comprising measurements of airflow, snoring, chest and abdominal wall movements, electrocardiography (ECG), position, and oximetry. They used it in the sleep laboratory for the study. A small sample of 12 children, 3 to 6 years of age, underwent routine PSG and in-laboratory portable testing on consecutive nights using the portable system. The outcome showed a good sensitivity for detecting a respiratory distress index, but a poor specificity. Nocturnal laboratory polysomnography is considered the gold standard in children in whom there is a high suspicion of OSAS. It is performed monitoring modifications in the electroencephalogram during sleep as well as cardiorespiratory function, airflow, and nocturnal oximetry. It is indicated for quantifying the severity of the disease, for preoperative evaluation, for predicting post-operative complications and the persistence of sleep breathing disorders after treatment. An important aspect is that the compliance to this exam is different in children and in adults. In fact, there is an anxious component in adults that does not allow adequate sleep. The same does not occur in children. This is well explained in several studies which have evaluated the so-called night-to-night variability, and ended up with the result that, in children, one night PSG is adequate. [115,116,117,118]. 

There is another test used in children: Nap PSG. It records sleeping and breathing in laboratory conditions during daytime. It is a simple and reliable test with a high sensibility and specificity [119].

Drug-induced sleep endoscopy (DISE) is mainly used in the pediatric population for the correct approach to children with residual OSAS after surgery. The main goal is to analyze the type of obstruction at the anatomical site of potential collapse [120]. 

The anesthetic protocol represents a debated point. Inhalation therapy with halothane or sevoflurane was used at the beginning, but several alterations to the pharyngeal muscle tone have led to it being progressively substituted by intravenous drugs, such as propofol, ketamine with glycopyrrolate, remifentanil, and midazolam [120].

Chen et al. [121] evaluated the role of DISE in the diagnosis and consequent aimed treatment in children with small tonsils. Their conclusion was that DISE is also required to evaluate the necessity of combined adenotonsillectomy in patients with a finding of small tonsils at clinical examinations. They focused on the differences between awake endoscopy and DISE. The main one is the possible underestimation of collapse in wakefulness. Moreover, the oropharynx obstruction was better defined in DISE than in awake endoscopy. 

In conclusion, although DISE has a well-defined codification in adults, the same cannot be said for children. This could be considered a paradox considering that the first study published in the literature regarding sleep endoscopy was the one by Croft in 1990 in a pediatric population [122].

## 5. Conclusions 

Obstructive sleep apnea in children is a condition with a multi-factorial etiology. The study of each single risk factor and of the associated comorbidities as well as the continuous monitoring of the patients are therefore of fundamental importance in order to be able to plan and eventually modify the therapeutic process correctly.

## Figures and Tables

**Table 1:** Studies that have analyzed obesity as a pediatric OSA risk factor.

Study	Year	Type of Study	Patients Number	Age	Parameters Evaluated	Conclusions
Arens R et al. [18]	2018	Case-control study	44	12.5 ± 2.8	Anatomical findings in obese children affected by OSAS compared to the ones in obese children	Significant upper airway lymphoid hypertrophy in obese children with OSAS. Larger parapharyngeal fat in obese children with OSAS but not a direct association with severity of OSAS or with obesity
Su M. et al. [21]	2016	Epidemiological study	5930	3–11	Age and sex;	No positive correlation between OSA and BMI
					AHI;	
					Arousal index;	
					BMI;	
					Mallampati;	
					AT hypertrophy;	
					Nocturnal/daytime symptoms	
Xu Z. et al. [26]	2008	Case-control Study	198	10.3 ± 2.1	Age and sex;	Positive relation between OSAS and degree of obesity
					BMI;	
					Waist circumference;	
					Neck circumference;	
					Waist-to-Height Ratio;	
					Symptoms;	
					AHI, Obstructive Apnea Index, Central Apnea, MinSaO2;	
					AT hypertrophy	
Andersen I.G. et al. [30]	2019	Longitudinal study	62	13.4 ± 3.1	Age and sex;	AHI normalization in 44% of patients and positive correlation between BMI and AHI parameters
					BMI;	
					AT hypertrophy;	
					AHI;	
					Sleep time (hours);	
					ODI	

**Table 2:** Reproducibility of clinical grading of tonsillar size.

Grade	Description
0	No tonsils seen
I	In tonsillar fossa
II	Visible beyond anterior pillars
III	Extended ¾ of way to midline
IV	Completely obstructing airway (kissing tonsils)

**Table 3:** Studies that have analyzed adenoid and/or tonsil hypertrophy as a pediatric OSAS risk factor.

Study	Year	Type of Study	Patients n°	Age	Parameters Evaluated	Conclusions
Kurnatowski P. et al. [33]	2007	Case–control study	225	10–13	Age and sex;	Negative emotional effect of adenotonsillar hypertrophy induced obstructive sleep disordered breathing
					Total sleep time;	
					AHI, ODI;	
					AT grade sec. Friedman;	
					Spielberger test;	
					Capra and Pastorelli scale	
Brietzke S.E. et al. [34]	2006	Meta-analysis (14 studies)	28 (mean)	4.9 (pooled mean age)	Age;	AT effective in reducing severity of OSAS in majority of patients
					Pre AT AHI;	
					Post AT AHI;	
					Success of AT;	
Kang K.T. et al. [38]	2017	Meta-analysis (4 studies)	18.25 (mean)	8.3 ± 1.1 (mean)	Age;	Effectiveness of lingual tonsillectomy for children with OSA caused by lingual tonsil hypertrophy
					BMI;	
					Other comorbidities;	
					CT, RMN, DISE;	
					Preoperative AHI;	
					Postoperative AHI	
					Preoperative ODI;	
					Postoperative ODI	
Lee C.F. et al. [41]	2016	Meta-analysis (11 studies)	11 (mean)	3.7 (mean)	Age;	Effectiveness in reducing AHI and MinSaO2, but complete resolution not achieved in most cases
					BMI;	
					Preoperative AHI;	
					Postoperative AHI	
					Preoperative ODI;	
					Postoperative ODI	

**Table 4:** Studies that have analyzed allergic rhinitis as a pediatric OSA risk factor.

Study	Year	Type of Study	Patients n°	Age	Parameters Evaluated	Conclusions
Cao Y. et al. [51]	2018	Meta-analysis (44 studies)	6086 total patients	47.97 (adults) 7.73 (children)	Age and Sex;	Children with OSA suffering from a higher incidence of AR. OSA adults with AR do not have any influences on sleep parameters
					BMI;	
					Neck circumference;	
					AHI;	
					ESS;	
					AR prevalence	
Kheirandish-Gozal L. et al. [52]	2014	Retrospective review	3071	2–14	Age and Sex;	Effective alternative to adenotonsillectomy, particularly in younger and non-obese Children
					BMI;	
					Pretreatment: AT grade;	
					Mallampati;	
					Total sleep time;	
					AHI, ODI;	
					Posttreatment: Pretreatment: AT grade;	
					Mallampati;	
					Total sleep time;	
					AHI, ODI	
Kheirandish-Gozal L. et al. [53]	2016	Prospective randomized trial study	92	2–10	Age and Sex;	Beneficial effects (reduction of AHI and ODI) in 71% of children treated with montelukast. No changes in those treated with placebo.
					BMI;	
					Pretreatment: AT grade;	
					Mallampati;	
					Total sleep time;	
					AHI, ODI;	
					Posttreatment: Pretreatment: AT grade;	
					Mallampati;	
					Total sleep time;	
					AHI, ODI	
Brouillette R.T. et al. [54]	2001	Triple-blind randomized placebo-controlled trial	25	1–10	Age and Sex;	Decrease in AHI/ODI values in 12/13 of fluticasone group. No changes in placebo group
					Pretreatment:	
					AHI, ODI;	
					Total sleep time;	
					AT grade;	
					AR symptoms	
					Posttreatment: AHI, ODI;	
					Total sleep time;	
					AT grade;	
					AR symptoms	

**Table 5:** Studies that have analyzed craniofacial abnormalities and genetics as a pediatric OSA risk factor.

Study	Year	Type of Study	Patients Number	Age	Parameters Evaluated	Conclusions
Follmar A. et al. [58]	2014	Retrospective cohort study	118	1 day–15 years	RDI;	Multifactorial etiology of RSD in children affected by Prader–Willi Syndrome
					Laryngomalacia;	
					macroglossia,	
					AT hypertrophy;	
					GERD.	
Onodera K. et al. [62]	2005	Case–control study	30	3.8 ± 1.4 (20) 7.9 ± 3 (10)	Questionnaire items:	Significant presence of RSD in patients affected by achondroplasia (AP)
					Snoring;	
					AHI;	
					Mouth breathing;	
					Occlusion;	
					Height and weight;	
					Ages of the eruption of deciduous teeth	
Pavone M. et al. [66]	2015	Retrospective study	88	1–14.5	Anthropometric data;	No correlations between MOAHI and age or BMI, positive correlations between MOAHI and Sp02
					BMI;	
					MOAHI, RDI, SpO2	
Guilleminault C. et al. [67]	2013	Retrospective study	34 patients	26.55	Clinical evaluation;	Commonly unrecognized abnormal breathing and its correlation with daytime fatigue and poor sleep in Ehlers–Danlos patients
					Rhinomanometry;	
					AHI, RDI, Sa02	
Kalaskar R et al. [69]	2012	Case report study	1	11 years old boy	Anatomical finding; orthodontic conformation	Association between Ellis–van Creveld syndrome and OSA
Cardiel Rios S.A. et al. [71]	2016	Case report study	1	10 years old boy	Anatomical finding; orthodontic conformation	Association between Noonan syndrome, malocclusion and OSA
Saxby C. et al. [74]	2018	Retrospective study	65	Not specified	Patients demographics; Type of midface advancement;	Positive outcomes after midface advancement in patients with craniosynostosis
					Preoperative: AHI, RDI, SaO2;	
					Postoperative: AHI, RDI, SaO2;	
					Blood pressure;	
Villa M.P. et al. [77]	2002	Randomized controlled study	32	4–10	Brouillette questionnaire;	Improved respiratory symptoms in patients who underwent oral appliance treatments
					physical examinations:	
					AHI, RDI, SaO2	

**Table 6:** Studies that have analyzed inflammatory factors and biomarkers as pediatric OSAS risk factors.

Study	Year	Type of Study	Patients n°	Age	Parameters Evaluated	Conclusions
Gozal D. et al. [59]	2007	Prospective study	355	5–7	AHI, RDI, SaO2;	Positive correlation between APOE epsilon4 allele and OSA and neurocognitive deficits
					Neurocognitive tests;	
					Blood draw.	
Khalyfa A. et al. [60]	2011	Case–control study	140	<6	ESS questionnaire;	Positive correlation between high TNF- α levels and OSAS
					AHI, RDI, SaO2M;	
					Serum TNF- α	
Tam C.S. et al. [79]	2006	Case–control study	113	7.3 ± 3.7	C-reactive protein;	Significantly elevated IFN-gamma levels and elevated IL-8 levels
					Cytokines: IL-1beta, IL-2, IL-4, IL-6, IL-8, IL-10, IL-12, GM-CSF, IFN-gamma and TNF-alpha.	
Gozal et al. [80]	2008	Case–control study	40	6.5 ± 0.7	Age and sex;	Higher levels of IL-6 and IL-10
					Ethnicity;	
					BMI:	
					AHI, RDI;	
					IL-6, IL-10.	
Park C.S. et al. [85]	2014	Case–control study	67	6 (3–16)	Age and sex;	High level of serum alpha amilase in severe OSAS children compared to moderate and mild ones and to the control group
					BMI;	
					AHI, RDI;	
					OSA-18 questionnaire;	
					Alpha amilase levels.	
Gozal D. et al. [86]	2009	Case–control study	60	6.6 ± 0.7	Clinical questionnaires;	Consistent alterations in urinary concentrations of specific protein clusters in OSA patients
					Height and weight;	
					BMI;	
					AHI; RDI, SaO2;	
					Urine collection	

## Abbreviations

AASM	American Association Sleep Medicine
AHI	Apnea-Hypopnea index
AT	Adenotonsillectomy
AR	Allergic Rhinitis
BMI	Body Mass Index
CPAP	Continuous Positive Airway Pressure
CRP	Reactive C- Protein
DISE	Drug Induced Sleep Endoscopy
ERS	European Respiratory Society
ESS	Epworth Sleepiness Scale
ICSD-3	International Classification of Sleep Disorders
ODI	Oxygen Desaturation Index
OSA	Obstructive sleep apnea
OSAS	Obstructive sleep apnea syndrome
PSG	Polysomnography
PSQ	Pediatric Sleep Questionnaire
RDI	Respiratory Disease Index
REM	Rapid Eye Movement
RERAs	Respiratory Effort Related Arousal
RSD	Respiratory Sleep Disorder
SSSDR	Sleeping Sleepless Sleepy Disturbed Rest questionnaire
